# Effectiveness and cost-effectiveness of an internet intervention for family caregivers of people with dementia: design of a randomized controlled trial

**DOI:** 10.1186/1471-244X-13-17

**Published:** 2013-01-10

**Authors:** Marco M Blom, Judith E Bosmans, Pim Cuijpers, Steve H Zarit, Anne Margriet Pot

**Affiliations:** 1 Department of Scientific Research, Alzheimer Nederland, Bunnik, Netherlands; 2 Department of Health Sciences, Faculty of Earth and Life Sciences, VU University, Amsterdam, Netherlands; 3 Department of Clinical Psychology, Institute for Research in Extramural Medicine, VU University, Amsterdam, Netherlands; 4 Department of Human Development and Family Studies, Pennsylvania State University, Altoona, PA, USA; 5 Trimbos-institute, Netherlands Institute of Mental Health and Addiction, Utrecht/Department of Clinical Psychology, Institute for Research in Extramural Medicine, VU University, Amsterdam, Netherlands

**Keywords:** Alzheimer’s disease, Informal caregivers, CBT, Psycho-education, Internet therapy, Mental health, Depression, Anxiety

## Abstract

**Background:**

The number of people with dementia is rising rapidly as a consequence of the greying of the world population. There is an urgent need to develop cost effective approaches that meet the needs of people with dementia and their family caregivers. Depression, feelings of burden and caregiver stress are common and serious health problems in these family caregivers. Different kinds of interventions are developed to prevent or reduce the negative psychological consequences of caregiving. The use of internet interventions is still very limited, although they may be a cost effective way to support family caregivers in an earlier stage and diminish their psychological distress in the short and longer run.

**Methods/design:**

A pragmatic randomized controlled trial is designed to evaluate the effectiveness and cost-effectiveness of ‘Mastery over Dementia’, an internet intervention for caregivers of people with dementia. The intervention aims at prevention and decrease of psychological distress, in particular depressive symptoms. The experimental condition consists of an internet course with 8 sessions and a booster session over a maximum period of 6 months guided by a psychologist. Caregivers in the comparison condition receive a minimal intervention. In addition to a pre and post measurement, an intermediate measurement will be conducted. In addition, there will be two follow-up measurements 3 and 6 months after post-treatment in the experimental group only. To study the effectiveness of the intervention, depressive symptoms are used as the primary outcome, whereas symptoms of anxiety, role overload and caregiver perceived stress are used as secondary outcomes. To study which caregivers profit most of the internet intervention, several variables that may modify the impact of the intervention are taken into account. Regarding the cost-effectiveness, an economic evaluation will be conducted from a societal perspective.

**Discussion:**

This study will provide evidence about the effectiveness and cost-effectiveness of an internet intervention for caregivers. If both can be shown, this might set the stage for the development of a range of internet interventions in the field of caregiving for people with dementia. This is even more important because future generations of caregivers will be more familiar with the use of internet.

**Trial registration:**

NTR-2051/RCT-DDB

## Background

In a recent report on the global economic impact of dementia [1], costs of dementia are estimated to be slightly over US$600 billion pro year worldwide. Costs include direct medical costs, direct costs of social care and costs attributed to informal care. As a consequence of the growing number of dementia patients, costs will rise in the future. Alzheimer’s Disease International has tentatively estimated an 85% increase in costs in 2030, based only on predicted increases in the number of people with dementia. As a consequence, there is a strong plea to invest in research and the development of cost effective approaches that meet the needs of people with dementia and their caregivers across the course of the illness.

Most people with dementia live at home and are cared for by their spouses, children or other family members. These caregivers play a crucial role in the quality of life and quality of care of their family members. Caring for a spouse with dementia can be a highly stressful experience associated with negative caregiver outcomes such as high rates of burden, psychological morbidity, depression and poorer immune function [2,3]. Different kinds of interventions have been developed for caregivers of people with dementia [4-7]. The majority of these interventions consists of psycho-educational programs providing information on dementia and the consequences for daily living, how to cope with related problems, which services are available and how to get help.

Research has shown that particular types of interventions are effective. One of the main characteristics of effective interventions is a psychological rather than purely educational approach [8]. Psychological treatment is aimed at improving caregivers’ abilities to cope with problem behavior or stressful situations or to enhance communication skills and asking support from others [9]. A recent review shows that psychological therapies and psychosocial interventions may be a potentially cost effective approach to improve outcomes and quality of life in both people with dementia and their family caregivers [10].

Several meta-analysis of randomized controlled trials on the effectiveness of internet interventions for depression or anxiety focusing on other target groups such as younger adults with depression, show that interventions are effective [11-14], even more if therapist support is part of the intervention [15]. There is also growing evidence that internet-based interventions are cost effective [16]. Family caregivers may favor internet interventions instead of meeting in a group or meeting with a professional face-to-face, due to lack of time or preferences concerning privacy. Caregivers may also be unwilling to visit a mental health care institute for themselves because in their view they are not the ones who need help. Given the growing internet sophistication of middle aged and older adults, it is expected that the number of potential users of internet interventions will grow substantially.

Until recently internet-based interventions were not developed for family caregivers of people with dementia. In November 2008, the innovative eMental Health intervention for family caregivers of people with dementia, called ‘Mastery over Dementia’, was launched in the Netherlands (for an English demo: http://www.masteryoverdementia.com). In this paper the design of the RCT to evaluate the effectiveness and cost-effectiveness of ‘Mastery over Dementia’ for caregivers of people with dementia will be described. The specific research questions are:

1. Is ‘Mastery over Dementia’ superior compared to ‘care as usual’ in terms of a clinically significant reduction in depressive symptoms, symptoms of anxiety, role overload and caregiver perceived stress?

2. Are effects maintained up to six months after ending the internet intervention?

3. Which caregivers respond best to the intervention compared to others?

4. Is the internet intervention cost effective from a societal perspective compared to the comparison group?

## Methods and design

To evaluate the effectiveness of ‘Mastery over Dementia’, we carry out a pragmatic randomized control trial (RCT). In the RCT there are two parallel groups. The experimental condition in which caregivers take part in the intervention (Mastery over Dementia) will be compared with a comparison condition in which caregivers receive a ‘minimal intervention’. The study protocol, information brochure and informed consent procedure were approved by the Medical Ethics Committee (METIGG, CCMO: NL27434.097.09).

### Recruitment

Participants are recruited in several ways: the website itself, the monthly digital newsletter of the Alzheimer’s Society in the Netherlands, leaflets at meetings of Alzheimer Cafes and information letters to memory clinics and other relevant care institutions. Participants are invited to fill in a first short questionnaire on the website. This questionnaire serves as a screening tool on the basis of which can be decided if participants are suitable for treatment. Caregivers will be included with a score > 4 on the Center for Epidemiological Studies-Depression scale, or a score > 3 on the HADS-A, or a minimum score of 6 on a one item burden scale ranging from 0-10. Caregivers with high scores on the CES-D and the HADS-A or having suicidal thoughts, were first contacted by an elderly care physician.

### Randomization

Randomization into two groups (experimental group and comparison group) takes place after assessment. Before randomization participants are stratified on the basis of two factors (sex: male/female and relationship: spouse/other). As a method for randomization block randomization with variable sizes is used. The allocation schedule is made with a computerized random number generator by an independent researcher and is unknown to the investigators of this study.

### Interventions

#### Experimental condition

The intervention ‘Mastery over Dementia’ was developed by the Netherlands Institute of Mental Health and Addiction in collaboration with a specialized Dutch health care provider (Geriant) and the Alzheimer’s Society in the Netherlands (Alzheimer Nederland) [17]. The program offered is a combination of evidence-based support strategies: psycho-education, cognitive behavioral therapy, problem solving therapy and assertiveness training. Relaxation is also incorporated. During the intervention, caregivers are supported on-line by a psychologist (coach) who gives feedback on the exercises sent by the caregivers. The coach has been trained in cognitive behavioral therapy.

‘Mastery over Dementia’ consists of eight lessons and a booster session (follow-up). Each lesson consists of information, practice rehearsal and some homework. Themes covered in the course are, for example:

– coping with behavioral problems

– arranging help from others

– non-helping en helping thoughts

– communicating problems

#### Comparison group

Caregivers in the comparison group receive a minimal intervention. There is no contact with a coach. The intervention consists of eBulletins with practical information about caring for someone with dementia. The bulletins are sent by mail according to a fixed schedule. Topics of the bulletin do not overlap with the content of ‘Mastery over Dementia’. Topics covered in the bulletins include:

– Driving and dementia

– Holiday breaks

– Medication and dementia

– Activities throughout the day

– Grieving and dementia

– Safety measures in the home

### Measures and measurement

There are 3 measurements in both groups. The first measurement is at baseline (pretest). The second measurement takes place halfway during the intervention course (after the fourth lesson) or after the receipt of the fourth bulletin (comparison group). This is approximately at 3 months after baseline. The third measurement (post-test) is conducted directly after the intervention (about 6 months after baseline). Additional measurements for the experimental group are at three months (9 months after baseline) and six months after completion (12 months after baseline). All measurements are self-report measures and are administered through the Internet. In both conditions automated emails are sent to participants asking them to fill in the questionnaires. This is repeated when participants do not respond within a week, and for a second time after two weeks. If participants do not answer a research assistant contacts them by phone.

#### Outcome measures in the RCT

For an overview of the instruments see Table 1.

**Table 1:** Overview of instruments (measures)

	**Pre-test**			**Post-tests**		
	**Intake**	**T = 1**	**T = 2**	**T = 3**	**T = 4**	**T = 5**
Outcome measures in the RCT						
-* Depressive symptoms: CES-D*	X		X	X	X	X
- *Anxiety symptoms: HADS-A*	X		X	X	X	X
- *Role overload: SPPIC*		X	X	X	X	X
- *Caregiver perceived stress: RMBPC*		X		X	X	X
Confounders/Modifying variables						
- *Perceived control: Mastery Scale*		X		X	X	X
- *Sense of competence: SSCQ*		X		X	X	X
- *Behavioral problems: RMBPC*		X		X	X	X
- *Demographic variables*	X					
- *Diagnosis/Duration of symptoms*	X					
- *Functional status of person with dementia: IQCODE*	X					
Outcome measures in the CEA						
- *Quality of life: EQ-5D+C*		X		X	X	X
- *Health care utilization: TIC-P*		X		X	X	X
- *Time spent on caregiving*	X			X	X	X

*CES-D* Centre for Epidemiological Studies Depression scale.

*EQ-5D+C* EuroQol 5 Dimensions plus Cognition.

*HADS-A* Hospital Anxiety and Depression Scale, Anxiety subscale.

*IQCODE* Informant Questionnaire on Cognitive Decline in the Elderly.

*RMBPC* Revised Memory and Behavioral Problem Checklist.

*SPPIC* Self Perceived Pressure from Informal Care scale.

*SSCQ* Short Sense of Competence Questionnaire.

*TIC-P* iMTA Questionnaires on Costs Associated with Psychiatric Illness.

##### Primary outcome measure: depressive symptoms

Depressive symptoms are measured with the Centre for Epidemiological Studies Depression scale (CES-D). The CES-D is a widely used self-report measure for the screening of depressive symptoms, and has also been used in research on caregiving. The Dutch version of the CES-D [18] is used in this study. It consists of 20 items for which subjects rate the frequency of symptoms during the past week. Scores range from 0 (rarely or none of the time present [less than 1 day]) to 3 (most or all of the time present [5-7 days]). The total score range is 0–60. Items represent major components of depressive symptomatology such as depressed mood, feelings of guilt and worthlessness, feelings of helplessness and hopelessness, psychomotor retardation, loss of appetite, and sleep disturbance. A shortened version of the CES-D on internet (not used in this study) was found to be reliable in an adult population [19].

##### Secondary outcome measure: anxiety symptoms

The 7-item anxiety subscale (Dutch version) of the Hospital Anxiety and Depression Scale [20] is used to measure the severity of anxiety symptoms. The Hospital Anxiety and Depression Scale (HADS) is an extensively used, brief self-report screening scale to investigate the prevalence of depression and anxiety symptoms. The anxiety subscale consists of 7 items rated on a scale ranging from 0 (not at all) to 3 (a great deal of the time). Total score ranges from 0 to 21 with higher scores indicating more anxiety. The Dutch version of the HADS shows good reliability in normal and clinical Dutch samples [21].

##### Secondary outcome measure: role overload

Feelings of role overload are measured by means of the Self Perceived Pressure from Informal Care scale (SPPIC) [22]. The SPPIC consists of 9 items, answers vary from 0 (totally disagree) to 4 (totally agree). The SPPIC is a one-dimensional hierarchical scale; higher scores mean more perceived pressure. The items are summed on the basis of dichotomized scores (cut off: totally disagree and disagree = 0; other answers = 1). Total scores range from 0 – 9. The original study [23] shows that reliability is good and that perceived pressure did not differ for male and female caregivers, and for spouse and non-spouse caregivers.

##### Secondary outcome measure: caregiver perceived stress

The Dutch version [24] of the Revised Memory and Behavioral Problem Checklist (RMBPC) [25] is used for measuring caregiver perceived stress. The questionnaire lists 24 items reflecting a variety of problems caregivers can encounter. Respondents have to rate the frequency of the occurrence of the specific behavior from 0 (never) to 4 (always). Each item rated with a score of 1 (seldom) of higher, is presented again raising the question how much people are stressed by the occurrence of the behavior (4-point scale from not at all stressed to very much stressed). The Caregiver Reaction Score is calculated as a mean score for all items together and additionally can be divided in separate reaction scores on depressive behavior (9 items), disruptive behavior (8 items) and memory-related behavioral problems (7 items). Overall scale reliability is good [24].

#### Additional measures in the RCT

##### Perceived control

Perceived control of events and ongoing situations will be assessed using a translated and abbreviated 5-item version of the Pearlin Mastery Scale. The original scale has 7 items regarding how much an individual perceives having control over things in his or her life. The scores per item vary from 0 (totally disagree) to 4 (totally agree). The Mastery Scale [26] has good psychometric properties. Compared to the original questionnaire, in the abbreviated version two items phrased in a positive way (‘I can do just about anything I really set my mind to’ and ‘What happens to me in the future mostly depends on me’) are left out. This abbreviated version shows good reliability [27]. Items are summed for a total mastery score (ranges from 0 to 20) with lower scores indicating greater perceived control.

##### Sense of competence

Caregiver’s sense of competence will be assessed by means of the Short Sense of Competence Questionnaire (SSCQ) [28]. The SSCQ consists of 7 items. Responses are rated on a 5-point scale with possible answers, ranging from totally agree (0) to totally disagree (4). The questionnaire has three dimensions: consequences for personal life of the caregiver (2 items), satisfaction with role of the caregiver themselves (2 items) and satisfaction with role of the person with dementia (3 items). The items are summed on the basis of dichotomized scores (cut off: totally disagree and disagree = 1; other answers = 0). Total score range from 0 – 7.

##### Frequency of reported behavioral problems

The 24 items of the Revised Memory and Behavioral Problem Checklist (RMBPC) [25] are used to make an inventory of the number of problems family caregivers encounter. Respondents have to rate from 0 to 4 the frequency of the occurrence of the specific behavior (0 = never; 1 = seldom; 2 = regularly; 3 = often; 4 = always). The total number of behaviors problems (0 – 24) will be calculated as well as the total mean score (Behavior Frequency Score). The Behavior Frequency can be subdivided into scores on Depression (9 items), Disruptive behavior (8 items) and Memory-related problems (7 items).

Other variables that may act as confounders or modifying variables will be measured. This concerns the following demographic characteristics of caregivers: sex, age, relationship (spouse, child or other), level of education and living arrangement (living together with care recipient). Relevant patients characteristics are: sex, age, duration of symptoms (as perceived by caregiver), functional status (assessed by means of a Dutch translation of a short version of the IQCODE [29]) and living condition (independently or within care facility).

#### Outcome measures in the economic evaluation

For an overview of the instruments see Table 1.

##### Quality of life

Quality of life will be measured using the 6-item self report instrument EQ-5D+C [30]. The EQ-5D+C is an extended version of the EuroQol [31] which measures health related quality of life and consists of five dimensions (mobility, self-care, main activity, pain and mood). The EQ-5D+C adds ‘cognition’ as a sixth dimension. Each dimension is rated as causing 'no problems', 'some problems' or 'extreme problems'. The EuroQol valuations appear to have good test-retest reliability [32]. QALYs will be calculated using the Dutch EuroQol tariff to estimate utilities [33].

##### Health care utilization

An adapted version of the iMTA Questionnaires on Costs Associated with Psychiatric Illness (TIC-P) will be used to measure health care utilization and productivity losses [34]. In this study both the direct costs related to the person with dementia with dementia and the direct costs related to the situation of the family caregivers themselves are measured. Direct costs will include the number of consultations with health care providers (e.g. general practitioner, physical therapist, psychologist, medical specialist, etcetera), medication, and admissions to hospitals and other institutions. Indirect costs are defined as the productivity lost due to absenteeism from paid and unpaid work and reduced efficiency at work (presenteeism).

##### Time spent on caregiving

This is measured by four questions tapping several main areas of caregiving (household tasks, personal hygiene, transportation outside, finances and administration). Caregivers are asked to estimate to total numbers of hours in the past week. In addition to this we also collect information on the availability of other informal caregivers and volunteers and the amount of time these others spent on caregiving for the person with dementia. Total amount of time the family caregiver spends on providing care and also the total amount of time spent by other informal caregivers or volunteers is calculated.

### Sample size

Symptoms of depression are the primary outcome measures and are used as starting point for the power calculations. A standardized effect size (Cohen's *d*) of 0.50 is considered a relevant treatment effect. Assuming an alpha of 0.05 and a statistical power (1-Beta) of 0.80 in a two-tailed test, we need 63 respondents in each of the conditions. Overall, we need 126 respondents to complete the study in total. Because we expect a minimum of 25 percent of caregivers to drop out during the data collection, we will recruit at least 175 caregivers. The numbers are calculated in Stata 7.0 (StataCorp 2001).

### Statistical analysis

All analyses in the RCT-study will be conducted according to the intention to treat principle. Missing data on follow-up measurements will be imputed using regression imputation. To examine differences between the outcomes for experimental and comparison group, paired t-tests will be conducted and estimates of effect sizes will be calculated. By using multiple regression analyses, we can correct for possible confounders. With a ‘generalized estimated equations’ (GEE) analysis the research question regarding the maintenance of the effectiveness of the intervention will be answered. In addition, profiles of caregivers who respond best to the intervention will be described by means of a multiple regression analysis which also contains modifying variables (modifier analysis). All analyses will be conducted using SPSS for Windows, version 19.

#### Economic analyses

This economic evaluation will involve both a cost-effectiveness analysis (CEA) and a cost-utility analysis (CUA). Incremental cost-effectiveness ratios will be calculated by dividing the difference in total costs between conditions by the difference in average effect size. Because we calculate costs over a period of six months, correction for inflation is not necessary. Missing data on follow-up measurements will be imputed using multiple imputation. Bias-corrected and accelerated bootstrapping with 5000 replications will be used to calculate 95% confidence intervals around the mean difference in total costs between the treatment groups. Bootstrapping will also be used to estimate the uncertainty surrounding the ICERs which will be graphically presented on cost-effectiveness planes. Cost-effectiveness acceptability curves and net monetary benefits will also be calculated. Cost-effectiveness acceptability curves show the probability that collaborative care is cost effective in comparison with usual care for a range of different ceiling ratios thereby showing decision uncertainty. Sensitivity analyses will be carried out to ascertain the robustness of the findings under different scenarios, e.g. under varying values of key-variables.

## Discussion

This study assesses the effectiveness and cost-effectiveness of an internet intervention for family caregivers of people with dementia. In the context of the fast growing number of people with dementia in years to come, there is an urgent need to develop cost-effective approaches that meet the needs of people with dementia and their family caregivers across the course of the illness. Some family caregiver may apply for help in the beginning of the course of the illness, others may use the intervention in a later stage. The potential effect of the internet intervention may act immediately or help people to keep on caring and prevent them from developing (more) health problems.

The RCT-study is conducted in the practical setting of a health provider who is specialized in dementia care and offers case management and a variety of support strategies for persons with dementia and family caregivers. Because internet delivered intervention is highly structured and leaves little room for the therapist to adapt it, in practice the structure remains intact without major adaptation [35]. Important advantage of such a pragmatic study is that results of the study have a high level of generalizibility.

As is the case with internet therapy (or research studies in this domain) in general [36], we expect participants to be more highly educated, with a high proportion of them having a university degree. It is also rather common that clients drop out of (research into) internet therapy. In a recent review on dropout from internet-based treatment for psychological disorders an average rate of 31% was found [37]. Evidence on any specific variables that may make an individual more likely to drop out is currently limited [37]. In this study we expect some family caregivers to drop out for specific disease related reasons like admission to an nursing home or death of the person with dementia. All together this may have a negative effect on the generalizability.

Primary outcome measure in the RCT is severity of depressive symptoms of the family caregiver. Anxiety symptoms, feelings of burden and caregiver stress are secondary outcomes. In a review article about consensus on outcome measures for psychosocial intervention research in dementia care, some recommendations are made for use of specific measures [38]. It is suggested that if depression is the primary intervention target, the CES-D has potential (although it requires further validation across Europe). Also the HADS is mentioned as an useful instrument. With regard to measuring feelings of burden, authors recommend the use of the Zarit Burden Interview (ZBI). This scale has been extensively used in intervention studies, however, only a few studies show significant changes on this scale. In this study we used the Self Perceived Pressure from Informal Care scale (SPPIC) which focuses on the aspect of role overload. To measure caregiver perceived stress we use the Revised Memory and Behavioral Problem Checklist (RMBPC).

In contrast to the CES-D and the HADS, both the SPPIC and RMBPC are not often used in a web-based manner. This means that scale properties like reliability may be influenced. However, research has shown that in the case of two scales measuring depression no statistically significant differences between paper and internet-based administration were found [39]. It is recommended that format of administration should not be changed when repeated measurements are made. Main advantage of web-based administration [40] is completeness and reliability of data (no missing values, no data entry errors). Other advantages are time flexibility for the family caregivers and low costs.

In conclusion, we expect that this study can add important information to the knowledge base on the effectiveness and cost-effectiveness of interventions to reduce psychological symptoms in informal caregivers of dementia persons with dementia. Given the steep rise in the number of people with dementia and the crucial role family caregivers play in caring for them, interventions that reach family caregivers at an early stage are essential [41].

## Competing interests

The authors declare that they have no competing interests.

## Authors’ contributions

MB co-ordinates the study, helped designing the study and wrote the manuscript. AMP is principal investigator and wrote the design of the study. SZ, JB and PC are members of the project group. All authors read and approved the final manuscript.

## Pre-publication history

The pre-publication history for this paper can be accessed here:

http://www.biomedcentral.com/1471-244X/13/17/prepub

## References

[B1] Alzheimer Disease International World Alzheimer Report: The global economic impact of dementia 2010 London, ADI

[B2] Brodaty H Donkin M Family caregivers of people with dementia Dialogues Clin Neurosci 2009 11 217 28 19585957 10.31887/DCNS.2009.11.2/hbrodaty PMC3181916

[B3] Braun M Scholz U Bailey B Perren S Hornung R Martin M Dementia caregiving in spousal relationships: a dyadic perspective Aging Ment Health 2009 13 426 436 10.1080/13607860902879441 19484607

[B4] Brodaty H Green A Koschera A Meta-analysis of psychosocial interventions for caregivers of people with dementia J Am Geriatr Soc 2003 51 657 664 10.1034/j.1600-0579.2003.00210.x 12752841

[B5] Pinquart M Sorensen S Helping caregivers of persons with dementia: which interventions work and how large are their effects? Int Psychogeriatr 2006 18 577 595 10.1017/S1041610206003462 16686964

[B6] Gallagher-Thompson D Coon DW Evidence-based psychological treatments for distress in family caregivers of older adults Psychol Aging 2007 22 37 51 17385981 10.1037/0882-7974.22.1.37

[B7] Smits CH de Lange J Dröes RM Meiland F Vernooij-Dassen M Pot AM Effects of combined programs for people with dementia living at home and their caregivers: a systematic review Int J Geriatr Psychiatry 2007 22 1181 1193 10.1002/gps.1805 17457793

[B8] Zarit S Femia E Behavioral and psychosocial interventions for family caregivers Am J Nurs 2008 108 47 53 18797226 10.1097/01.NAJ.0000336415.60495.34

[B9] Selwood A Johnston K Katona C Lyketsos C Livingston G Systematic review of the effect of psychological interventions on family caregivers of people with dementia J Affect Disord 2007 101 75 89 10.1016/j.jad.2006.10.025 17173977

[B10] Olazarán J Reisberg B Clare L Cruz I Peña-Casanova J Del Ser T Woods B Beck C Auer S Lai C Spector A Fazio S Bond J Kivipelto M Brodaty H Rojo JM Collins H Teri L Mittelman M Orrell M Feldman HH Muñiz R Nonpharmacological therapies in Alzheimer's disease: a systematic review of efficacy Dement Geriatr Cogn Disord 2010 30 161 178 10.1159/000316119 20838046

[B11] Spek V Cuijpers P Nyklicek I Riper H Keyzer J Pop V Internetbased cognitive behaviour therapy for symptoms of depression and anxiety: a meta-analysis Psychol Med 2007 37 319 328 10.1017/S0033291706008944 17112400

[B12] Van Straten A Cuijpers P Smits N Effectiveness of a Web-Based Self-Help Intervention for Symptoms of Depression, Anxiety, and Stress: Randomized Controlled Trial J Med Internet Res 2008 10 e7 10.2196/jmir.954 18364344 PMC2483843

[B13] Cuijpers P Marks I van Straten A Cavanagh K Gega L Andersson G Computer-aided psychotherapy for anxiety disorders: a meta-analytic review Cogn Behav Ther 2009 38 66 82 10.1080/16506070802694776 20183688

[B14] Andrews G Cuijpers P Craske MG McEvoy P Titov N Computer therapy for the anxiety and depressive disorders is effective, acceptable and practical health care: a meta-analysis and pilot implementation PLoS One 2010 5 e13196 10.1371/journal.pone.0013196 20967242 PMC2954140

[B15] Andersson G Cuijpers P Internet-based and other computerized psychological treatments for adult depression: a meta-analysis Cogn Behav Ther 2009 38 196 205 10.1080/16506070903318960 20183695

[B16] Warmerdam L Smit F van Straten A Riper H Cuijpers P Cost-Utility and Cost-effectiveness of Internet-Based Treatment for Adults With Depressive Symptoms: Randomized Trial J Med Internet Res 2010 12 e53 10.2196/jmir.1436 21169166 PMC3057305

[B17] Pot AM Dorland L Grolleman J Riper H Blom M Vuister J Mastery over dementia: online counseling for family caregivers of people with dementia Int Psychogeriatr 2007 19 Suppl 11

[B18] Radloff LS The CES-D Scale: A Self-Report Depression Scale for Research in the General Population Applied Psychol Measur 1977 1 385 401 10.1177/014662167700100306

[B19] Donker T Van Straten A Marks IM Cuijpers P Brief self-rated screening for depression on the internet J Affect Disord 2010 122 253 259 10.1016/j.jad.2009.07.013 19679358

[B20] Zigmond AS Snaith RP The Hospital Anxiety and Depression Scale Acta Psychiatr Scand 1983 67 361 370 10.1111/j.1600-0447.1983.tb09716.x 6880820

[B21] Spinhoven P Ormel J Sloekers PP Kempen GI Van Hemert AM A validation study of the Hospital Anxiety and Depression Scale (HADS) in different groups of Dutch subjects Psychol Med 1997 27 363 370 10.1017/S0033291796004382 9089829

[B22] Pot AM Van Dyck R Deeg DJH Ervaren druk door informele zorg: constructie van een schaal Tijdschr Gerontol Geriatr 1995 26 214 219 8750982

[B23] Pot AM Deeg DJH van Dyck R Jonker C Psychological distress of caregivers: the mediator effect of caregiving appraisal Patient Educ Couns 1998 34 43 51 10.1016/S0738-3991(98)00048-2 9697556

[B24] Teunisse S Clinimetrics in Dementia. PhD thesis. University of Amsterdam, Department of Neurology of the Academic Medical Center 1997

[B25] Teri L Truax P Logsdon R Uomoto J Zarit S Vitaliano PP Assessment of behavioral problems in dementia: the revised memory and behavior problems checklist Psychol Aging 1992 7 622 631 1466831 10.1037//0882-7974.7.4.622

[B26] Pearlin LI Schooler C The structure of coping J Health Soc Beh 1978 19 2 21 10.2307/2136319 649936

[B27] Comijs HC Deeg DJH Dik MG Twisk JWR Jonker C Memory complaints; the association with psycho-affective and health problems and the role of personality characteristics; a 6-year follow-up study J Affect Disord 2002 72 157 165 10.1016/S0165-0327(01)00453-0 12200206

[B28] Vernooij-Dassen M Felling A Brummelkamp E Dautzenberg M van den Bosch G Grol R A short sense of competence questionnaire (SSCQ): measuring the caregiver's sense of competence J Am Geriatr Soc 1999 47 256 257 9988301 10.1111/j.1532-5415.1999.tb04588.x

[B29] Jorm AF A short form of the Informant Questionnaire on Cognitive Decline in the Elderly Psychol Med 1994 24 145 153 10.1017/S003329170002691X 8208879

[B30] Brooks R EuroQol: the current state of play Health Policy 1996 37 53 72 10.1016/0168-8510(96)00822-6 10158943

[B31] Group EQ Euroqol – a new facility for the measurement of health related quality of life Health Policy 1990 16 199 208 10109801 10.1016/0168-8510(90)90421-9

[B32] Van Agt HM Essink-Bot ML Krabbe PF Bonsel GJ Test-retest reliability of health state valuations collected with the EuroQol questionnaire Soc Sci Med 1994 39 1537 1544 10.1016/0277-9536(94)90005-1 7817218

[B33] Lamers LM Stalmeier PF McDonnell J Krabbe PF van Busschbach JJ Measuring the quality of life in economic evaluations: the Dutch EQ-5D tariff Ned Tijdschr Geneeskd 2005 149 1574 1578 16038162

[B34] Hakkaart-Van Roijen L Manual Trimbos/iMTA questionnaire for costs associated with psychiatric illness (in Dutch) 2002 Rotterdam: institute for Medical Technology Assessment

[B35] Andersson G Carlbring P Cuijpers P Internet interventions: moving from efficacy to effectiveness Electronic Journal of Applied Pschology 2009 5 18 24

[B36] Andersson G Bergström J Holländare F Carlbring P Kaldo V Ekselius L Internet-based self-help for depression: a randomised controlled trial Br J Psychiatry 2005 187 456 461 10.1192/bjp.187.5.456 16260822

[B37] Melville K Casey L Kavanagh D Dropout from Internet-based treatment for psychological disorders Br J Clin Psychol 2010 49 455 471 10.1348/014466509X472138 19799804

[B38] Moniz-Cook E Vernooij-Dassen M Woods R Verhey F Chattat R De Vugt M Mountain G O’Connell M Harrison J Vasse E Droës RM Orrell M A European consensus on outcome measures for psychosocial intervention research in dementia care Aging Ment Health 2008 12 14 29 10.1080/13607860801919850 18297476

[B39] Holländare F Andersson G Engström I A comparison of psychometric properties between internet and paper versions of two depression instruments (BDI-II and MADRS-S) administered to clinic patients J Med Internet Res 2010 12 e49 10.2196/jmir.1392 21169165 PMC3057311

[B40] Kongsved SM Basnov M Holm-Christensen K Hjollund NH Response rate and completeness of questionnaires: a randomized study of Internet versus paper-and-pencil versions J Med Internet Res 2007 9 e25 10.2196/jmir.9.3.e25 17942387 PMC2047288

[B41] Alzheimer Disease International World Alzheimer Report: The benefits of early diagnosis and intervention 2011 London, ADI

